# Developing a dementia-specific health state classification system for a new preference-based instrument AD-5D

**DOI:** 10.1186/s12955-017-0585-0

**Published:** 2017-01-25

**Authors:** Kim-Huong Nguyen, Brendan Mulhern, Sanjeewa Kularatna, Joshua Byrnes, Wendy Moyle, Tracy Comans

**Affiliations:** 1 Center for Applied Health Economics, Menzies Health Institute Queensland, Griffith University, Nathan, QLD 4111 Australia; 2 The NHMRC Cognitive Decline Partnership Centre, Sydney, Australia; 3 Centre for Health Economics Research and Evaluation, University of Technology Sydney, Sydney, Australia; 4 Centre for Health Practice Innovation, Griffith University, Brisbane, Australia; 5 Metro North Hospital and Health Service District, Brisbane, Australia

**Keywords:** Dementia, Health-related quality of life, Preference-based measure, Health state classification, QOL-AD, QALY, Economic evaluation

## Abstract

**Background:**

With an ageing population, the number of people with dementia is rising. The economic impact on the health care system is considerable and new treatment methods and approaches to dementia care must be cost effective. Economic evaluation requires valid patient reported outcome measures, and this study aims to develop a dementia-specific health state classification system based on the Quality of Life for Alzheimer’s disease (QOL-AD) instrument (nursing home version). This classification system will subsequently be valued to generate a preference-based measure for use in the economic evaluation of interventions for people with dementia.

**Methods:**

We assessed the dimensionality of the QOL-AD to develop a new classification system. This was done using exploratory and confirmatory factor analysis and further assessment of the structure of the measure to ensure coverage of the key areas of quality of life. Secondly, we used Rasch analysis to test the psychometric performance of the items, and select item(s) to describe each dimension. This was done on 13 items of the QOL-AD (excluding two general health items) using a sample of 284 residents living in long-term care facilities in Australia who had a diagnosis of dementia.

**Results:**

A five dimension classification system is proposed resulting from the three factor structure (defined as ‘interpersonal environment’, ‘physical health’ and ‘self-functioning’) derived from the factor analysis and two factors (‘memory’ and ‘mood’) from the accompanying review. For the first three dimensions, Rasch analysis selected three questions of the QOL-AD (‘living situation’, ‘physical health’, and ‘do fun things’) with memory and mood questions representing their own dimensions. The resulting classification system (AD-5D) includes many of the health-related quality of life dimensions considered important to people with dementia, including mood, global function and skill in daily living.

**Conclusions:**

The development of the AD-5D classification system is an important step in the future application of the widely used QOL-AD in economic evaluations. Future valuation studies will enable this tool to be used to calculate quality adjusted life years to evaluate treatments and interventions for people diagnosed with mild to moderate dementia.

## Research highlights

- This study proposes a new health state classification system for dementia based on the widely used health-related quality of life instrument QOL-AD.
- This is the first step toward the development of a new preference-based measure that improves the estimation of QALYs in people with dementia
- This will help decision makers make better resource allocation decisions relating to dementia treatment and management

## Background

Dementia is a progressive disorder of the brain that is common in older populations. The damage to brain cells as a consequence of dementia results in a loss of cognitive ability, and the ability to think, reason and function. This leads to a reduction in quality of life, for example by affecting daily activities, including the ability to take care of oneself. As the signs and symptoms of dementia become worse it also affects the lives and emotional wellbeing of families and carers [1, 2].

Given the aging of the population worldwide, the number of people with dementia is expected to double every 20 years, with the incidence of nearly 7.7 million new cases per year [3]. The estimated worldwide cost of dementia was US$604 billion in 2010, of which the most dominant component is informal and social care. This places dementia as the third most costly disease, just after cancer and cardiovascular disorders [4]. In Australia, an estimated 322,000 people had dementia in 2013 and this figure is projected to rise to almost 900,000 by 2050 [5, 6]. Dementia care is a significant financial burden on the healthcare sector and society, and will become the third largest source of health and residential aged care spending within two decades, with costs forecast to be approximately 1% of gross domestic product in Australia by 2030 [7]. Therefore it is imperative that new treatment methods and approaches to care are developed, and are cost effective.

Health care reimbursement agencies around the world use cost utility analysis to determine the cost effectiveness of new healthcare interventions. This approach uses Quality Adjusted Life Years (QALYs) as a single index measure of outcome that combines preference for both length of life and its quality [8, 9], from which cost-per-QALY ratios are calculated for the healthcare interventions. In a QALY calculation, health-related quality of life (HRQL) is usually used as it includes aspects of quality of life that are affected by a health condition [10]. The preference score (or “weight”) for HRQL used to generate QALY is usually measured on a scale of 0 (death) to 1 (full health), representing the preference (value) of different levels of health (i.e. health states). However, it can be negative if the preference suggests there are health states worse than death [9]. These weights are estimated using the preferences of the population for relevant health states which are elicited in health state valuations using techniques such as time trade off, standard gambles, and discrete choice experiments.

The QALY is widely used in economic evaluation of healthcare interventions because it represents a common unit of improvements (benefits) that enables comparison between interventions when clinical outcomes are not directly comparable. This comparative advantage is possible due to the critical assumptions that preferences is a valid value measure, which can be measured across individuals, aggregated and used for the group; and a QALY is a QALY regardless of who gains or loses it [11]. However, the QALY does not address the problem of comparing health and non-health outcomes because it only measures health-related quality of life (by construct), not social welfare [12].

The EuroQol five dimensions questionnaire (EQ-5D) [13] is the most widely used generic preference-based measure to provide utility values for use in the generation of QALYs. The EQ-5D measures HRQL in five dimensions (mobility, self-care, usual activities, pain/discomfort and anxiety/depression) with three response levels (none, some, extreme/unable). A five response level version (EQ-5D-5 L) has also been developed [14]. The EQ-5D is a generic preference-based instrument, meaning that it is intended to represent all relevant aspects of health regardless of disease area. However, its validity is questionable in some health conditions and in particular with regards to dementia. First, the descriptive system may not be sensitive to the HRQL impacts of particular conditions, meaning that interventions that improve these aspects are not considered cost effective. For example, cognition and social relationships are not explicitly captured by the EQ-5D [15] while these aspects are considered important to the HRQL for those with dementia [16, 17]. The absence of a cognitive component in the EQ-5D is a significant challenge when using the EQ-5D for diseases of the mind [18]. In addition, relationships with family and social support are important aspects of the HRQL for those with dementia, but is not measured with the EQ-5D [19]. Second, there is evidence that the EQ-5D has low validity as measurement tool, due to ceiling effects and little correlation with severity of dementia [18, 20]. Whilst a number of studies have reported good reliability with the EQ-5D in mild to moderate dementia conditions [21], known ceiling effects within the EQ-5D leads to difficulty in determining utility values for severe conditions [17–20]. Third, a recent study found there are substantial problems of validity between patient and proxy ratings. With the EQ-5D, different proxies have provided different ratings for the same patients’ health [18]. This is important in the field of dementia where proxies are often relied upon to complete surveys on behalf of the patient. Last but not least, there is evidence of mismatch between the EQ-5D and respondent generated attributes [22]. As such, the validity of the EQ-5D for use in resource allocation in dementia may be limited [23].

On the other hand, there are a number of dementia-specific HRQL instruments, such as quality of life in Alzheimer's disease (QOL-AD) [24, 25], dementia quality of life instrument (DQOL) [26], quality of life questionnaire for dementia (QOL-D) [27], and dementia-specific health-related quality of life instrument (DEMQOL) [28]. While these instruments are frequently used in studies exploring HRQL of people with dementia, unfortunately, these instruments are not preference-based and therefore cannot be used to calculate QALYs for economic evaluations. To deal with this deficit, there has been interest in the development of preference-based measure from dementia-specific instruments. The DEMQOL-U [29, 30], which was developed from the DEMQOL, is such an example. DEMQOL-U measures dementia-specific HRQL on five dimensions (positive emotion, cognition, negative emotion, relationships and loneliness) and has been demonstrated to have a similar validity to EQ-5D [29]. However, it has been suggested that DEMQOL-U may be limited as it does not directly measure physical health [31].

Arons et al. (2015) recently developed a 6-dimension preference-based instrument for dementia (DQI) that covers physical health along with mood, memory, self-care, social functioning and orientation. The health state values were derived from professionals working with people with dementia (*N* = 207) and respondents from the general population (*N* = 631), using a discrete choice experiment. However, further work is required on the validity of the DQI given that it was not developed from an existing psychometrically validated HRQL tool.

The QOL-AD is a valid HRQL instrument for use with people with mild to moderate dementia [32]. It is a brief-measure that is widely used in clinical trials and observational studies, and has been validated in at least ten countries with evidence of psychometric acceptability and sensitivity to psychosocial interventions [16]. A proxy version is recommended for those with severe dementia [33].

In this paper, we describe the development of a dementia-specific health state classification system based on the QOL-AD instrument. This is the first step toward a complete preference-based measure that can be used in economic evaluations of interventions for people with a diagnosis of dementia or cognitive decline (the second step involves a valuation study to develop a utility scale for use in the estimation of QALYs). This instrument will be called AD-5D and will be the first dementia-specific preference-based HRQL instrument with a value set based on the preferences of the Australian population that accepts condition-specific utility values for use in a resource allocation decision making system [34].

## Data and methods

### QOL-AD instrument

The QOL-AD was originally developed as a 13-item instrument designed to collect HRQL information from people with Alzheimer’s disease [24, 25]. It evaluates the patient’s physical condition, mood, interpersonal relationships, ability to participate in meaningful activities, and financial situation. These domains are considered important in cognitively impaired adults [16]. Each item is rated on a four-point scale: 1 = poor and 4 = excellent. Two of the 13 items are global measures: ‘self as a whole’ and ‘life as a whole’. An adaption with 15 items was developed for use in long-term care facilities (Table 1) [35]. This version shares ten items with the original version and includes five new items that assess patient relationships with staff, keeping busy, self-care, living with others, and making choices. The caregiver version was used in this study, with the specification that items appearing on both versions of the instrument would be included in the classification system. Each adaptation of the QOL-AD has two versions; one is completed by the patient (self-rated), and one by the caregiver (proxy-rated). When both patient and carer instruments are used, a weighted composite score is calculated by giving greater weight to the patient’s rating relative to the caregiver’s.

**Table 1:** Quality of life Alzheimer’s Disease (QOL-AD) instrument

Original	Long-term care adaptation	Short names
How do you feel about your physical health?	How do you feel about your physical health?	Physical health
How do you feel about your energy level?	How do you feel about your energy level?	Energy
How has your mood been lately?	How has your mood been lately?	Mood
How about your living situations? How do you feel about the place you live now?	How about your living situations? How do you feel about the place you live now?	Living situation
How about your memory?	How about your memory?	Memory
How about your family and your relationship with family members?	How about your family and your relationship with family members?	Family relationship
How do you feel about your marriage?		Marriage
	How do you feel about your relationship with people who work here?	Relationship with staff
How would you describe your current relationship with your friends?	How would you describe your current relationship with your friends?	Friendship
How do you feel about yourself when you think of yourself overall and all the different things about you?	How do you feel about yourself when you think of yourself overall and all the different things about you?	Self-overall
	How do you feel about your ability to keep busy?	Keep busy
How about your ability to do fun things that you enjoy?	How about your ability to do fun things?	Do fun things
	How do you feel about your ability to take care of yourself?	Take care of self
	How do you feel about your ability to live with others?	Live with other
	How about your ability to make choice in your life?	Make choice
How do you feel about your current situation with money, your financial situations?		Financial situation
When you think of your life overall, everything together, how do you feel about your life	When you think of your life overall, everything together, how do you feel about your life?	Life overall

### QOL-AD data

#### Participants

The sample with QOL-AD data consisted of 284 residents living in 35 long-term care facilities in South-East Queensland, Australia. Participants’ age ranged from 60 to 100 years. All participants had a diagnosis of dementia, of whom 32% had Alzheimer’s disease, 15% had vascular dementia, and the rest had other forms of dementia (including dementia with Lewis body, frontotemporal lobar degeneration, alcohol related dementia and other unspecified forms). Of the cohort, 76% were female, 57% older than 85 years of age and 11% had English as their second language.

#### Procedures

The long-term care adaption of the QOL-AD was administered to people living with dementia in nursing homes who were involved in a cluster randomised controlled trial that examined usual care with an interactive therapeutic robot and with a look-alike plush toy (without the robotic features) [36]. Of the 15 items, the two global HRQL items were administered but are not relevant for inclusion in a health state classification. Therefore, all data analyses were performed on the 13 individual items.

### Classification system development

Brazier and colleagues [37] have described a six stage process for developing a preference-based instrument from an existing condition-specific HRQL measure. This has previously been used to provide a basis for the derivation preference-based indices for dementia (DEMQOL-U) [29], cancer (EORTC-8D) [38], asthma (AQL-5D) [39], overactive bladder (OAB-5D) [40], and urinary incontinence (KHQ) [41].

The first four stages of this process involve the derivation of a health state classification system: dimension assessment (stage 1), item assessment and selection (stage 2), item level reduction (stage 3) and validation of the classification system (stage 4). The last two stages are a valuation survey (stage 5) and modelling health state values (stage 6) to develop the appropriate algorithm to obtain utility values for the preference-based measures [37].

This paper presents the first three stages of the process of developing a preference-based instrument from the QOL-AD.

### Dimensionality assessment (stage 1)

We conducted exploratory factor analysis to investigate the number of latent constructs (i.e. factors or dimensions) underlying the items, and the magnitude of correlation between items and each dimension. Factor extraction was conducted using promax (oblique) rotation which assumes that factors are related. The factor models were selected based on the eigenvalues, total variance, and the meaningfulness of the factors. We used factor loading above 0.3 as a cut-off point, as suggested in previous studies [37].

Confirmatory factor analysis was then performed for model selection. Fit statistics such as the comparative fit index and root mean square errors of approximation were compared across models. A comparative fit index greater than 0.90 and a root mean square errors of approximation lower than 0.05 indicate an acceptable model fit [42].

In the development of a condition specific preference-based measure, it is important to represent the key dimensions in the original measure as clearly as possible. However factor analysis may produce models that do not include a dimensionality structure with all of the key dimensions of the original measure. Therefore, the overall structure of the measure was assessed by the project team alongside the factor analysis to ensure that all of the key dimensions were included in the classification system structure.

### Item assessment and selection (stage 2) and item level reduction (stage 3)

Following the factor analysis, we conducted Rasch analysis for each identified factor. The purpose was to test and to eliminate items that did not perform well or accurately represent the dimension, and to select items to include in the classification system. This analysis was performed using RUMM2030 software [43].

#### The Rasch model

The Rasch model belongs to a class of item response theory statistical models. Item response theory takes the modern test theory approach, in which the focus is modelling the probability of a person’s response to an item as a function of the underlying trait and the item parameter. Item response theory is an improvement over classical test theory because it provides a statistical model of how and why individuals respond as they do to an item – and independently, about the items themselves. In practice, the Rasch model has been used for analyses of the psychometric properties of composite measures such as cognitive and personality traits, health outcomes, and HRQL where unidimensional constructs within the measures are assessed.

In Rasch analysis, a mathematical model is specified that provides the link between item scores and the hypothetical latent trait. It assumes that the probability of endorsing an item is a logistic function of the relative difference between item location (difficulty) and person location (ability) on a linear scale [44]. In other words, Rasch analysis assesses the performance of individual items in relation to the underlying trait. Details of the model and its advantages have been extensively described (see for example [45] and [46]).

The Rasch model is based on three major assumptions: unidimensionality, local independence, and invariance [44]. Unidimensionality means the (included) items measure a single underlying trait (e.g., physical functioning or social relationships). Local independence refers to the assumption that the trait is the sole influence on a person’s response to an item. Differential item functioning (or invariance) states that the estimation of item parameters is independent of the sample of respondents used to derive the estimate.

#### Criteria used to test item performance

In our analysis, these assumptions were investigated using the following indicators: response category ordering, item-fit and person-fit, differential item functioning, and person and item separation reliability [44].

When respondents are unable to distinguish between response categories (levels) for a particular item or the categories are not working as intended, the item exhibits response disordering. This was assessed by determining whether there was a monotonic increase across thresholds for each item. We merged adjacent response options for the disordered item or discarded them if other items were not disordered and perform comparably by other indicators.

Item and person fit was measured by three overall fit statistics. The person-fit and item-fit statistics with mean approximately zero and a standard deviation around unity indicate a good fit as these two statistics were transformed to approximate z-distributions. The item-trait interaction statistics follow a chi square distribution that reflects the property of invariance across that trait. An insignificant chi square indicates that the hierarchical ordering of items does not vary across the trait, suggesting a good fit [45]. A common cause for poor fit is that the items may measure another latent trait, leading to multidimensionality. Removal of misfitting items may restore unidimensionality. Outliers (respondents with unexpected or extreme responses) may also affect model fit at the item level. Removal of these outliers can make a significant difference to the dimension’s internal construct validity. During the analysis, where misfit was identified, we removed items causing multidimensionality until the Rasch model statistics showed an acceptable fit.

Differential item functioning can also affect model fit. This occurs when different groups within the sample (e.g., male versus female) respond in a different manner to an individual item, despite the similarity in the underlying characteristics being measured. Differential item functioning can be assessed by producing independent estimates of item location using subgroups of individuals. Here, we tested the differential item functioning by gender, age and whether or not English was the second language, the three available individual characteristics.

Item and person separation statistics indicate the spread of items and persons along the latent scale, and thus the discriminatory power of individual items. The person separation index differentiates individuals on the constructed scale, while the item separation index identifies item hierarchy [47]. Low person separation (<0.8) implies that the instrument may not be sensitive enough to distinguish between extreme responses. A sample with higher response variance and/or an instrument with more items may improve person separation index. Low item separation (<0.9) implies that the sample is not large enough to confirm the item construct validity of the instrument. The item separation index can be improved with a large item response range and/or a large sample of individuals.

## Results

Table 2 shows the summary statistics of QOL-AD scores for the 13 items. The overall amount of missing data varied across individual items: the lowest missing rate was attributable to item 1 (‘physical health’, 3%) and highest missing rate to item 13 (‘ability to make choices’, 19%). Most items were free of floor and ceiling effects, shown by the low proportion (less than 20%) of participants that answered the minimum and maximum score possible. Items 6 and 7 (relationship with family and staff) exhibited a slight ceiling effect. However, most responses fell into the middle response categories. The Cronbach's alphas for the total score (0.853) and for individual items indicate high internal consistency. The average inter-item correlations of all items are relatively low, suggesting the multidimensional property of the QOL-AD instrument.

**Table 2:** Summary statistics

		N (284)	% missing	Mean	Std. Dev.	Floor effect (% min score)	Ceiling effect (% max score)	Factor loading	Cronbach's alpha^a^
Item 4	Living situation	256	9.86	1.754	0.839	8.59	17.19	0.341	0.843
Item 6	Family	259	8.80	1.931	0.828	7.72	23.17	0.683	0.852
Item 7	Staff	247	13.03	1.899	0.766	4.45	20.24	0.380	0.850
Item 8	Friends	237	16.55	1.814	0.797	7.17	16.88	0.590	0.844
Item 12	Live with others	239	15.85	1.703	0.820	8.79	14.23	0.465	0.840
Item 13	Make choices	229	19.37	1.729	0.820	9.17	14.41	0.398	0.837
Item 1	Physical health	276	2.82	1.634	0.849	12.32	11.96	0.814	0.846
Item 2	Energy	273	3.87	1.516	0.862	14.29	10.26	0.626	0.839
Item 9	Keep busy	243	14.44	1.593	0.864	13.17	11.93	0.757	0.839
Item 10	Do fun things	238	16.20	1.542	0.917	15.13	14.29	0.757	0.836
Item 11	Self-care	243	14.44	1.765	0.822	7.41	17.28	0.396	0.843
Item 3	Mood	261	8.10	1.586	0.793	9.96	9.20	-	0.842
Item 5	Memory	263	7.39	1.430	0.866	16.35	8.75	-	0.843

*Abbreviations*: *Std. Dev.* standard deviation, *min* minimum, *max* maximum

^a^Cronbach’s alpha for individual item is calculated as the overall alpha when the item is excluded from the pool

### Dimensionality assessment (stage 1)

The exploratory and confirmatory factor analysis indicated that a three factor model fitted the data after excluding item 3 (mood) and item 5 (memory). ‘Memory’ did not load on any factor, which was likely due to the different construct measured by this item in comparison to all other items. ‘Mood’ was separated out as an individual dimension due to its face validity: it has been suggested that mood is a relevant HRQL domain for people with dementia [16, 17]. The remaining three factors were defined as ‘interpersonal environment’, ‘physical health’ and ‘self-functioning’. Rasch analyses were conducted based on these three dimensions.

### Item performance and selection (stage 2) and item level reduction (stage 3)

Tables 3 and 4 display the goodness of fit for the Rasch models and the item-by-item psychometric and Rasch analyses for the three dimensions identified by the factor analyses. The results for each of the three dimensions are described below.

**Table 3:** Goodness of fit to the Rasch model for each dimension (factor)

Dimensions	χ2 (df)	*p*-value	Item fit (SD)	Person fit (SD)	PSI
Interpersonal environment	37.9 (36)	0.830	0.064 (1.004)	−0.708 (1.590)	0.577
Physical health	10.9 (10)	0.358	0.666 (0.130)	−0.405 (0.749)	0.128
Self-functioning	4.1 (10)	0.941	0.015 (0.020)	−1.032 (1.310)	0.443

*Abbreviations*: *SD* standard deviation, *PSI* person separation index

**Table 4:** Rasch analysis results by individual items

		Response category ordering	Item range		Fit residuals	χ2 *p*-value	DIF
Interpersonal environment							
Item 4	Living situation	Ordered	−1.636	1.879	1.633	0.169	No DIF
Item 6	Family	Ordered	−1.428	2.012	−0.311	0.597	Age
Item 7	Staff	Ordered	−1.373	1.667	−1.481	0.247	No DIF
Item 8	Friends	Ordered	−1.634	1.932	0.207	0.977	No DIF
Item 12	Live with others	Ordered	−1.827	2.318	0.223	0.738	Age
Item 13	Make choices	Ordered	−1.978	2.467	0.113	0.951	ESL
Physical health							
Item 1	Physical health	Ordered	−1.183	1.691	0.758	0.441	No DIF
Item 2	Energy	Ordered	−1.282	1.801	0.574	0.288	Gender
Self-functioning							
Item 9	Keep busy	Ordered	−2.291	2.588	0.000	0.934	Age
Item 10	Do fun things	Ordered	−1.958	2.563	0.029	0.725	No DIF
Item 11	Self-care	Disordered (poor/fair)	-	-	-	-	-

*Abbreviations*: *DIF* differential item functioning

#### Interpersonal environment

All six items were ordered on the logit scale. These items fitted the model well in terms of fit residual and χ^2^ *p*-value. The person-item threshold distribution displayed slightly weak targeting at both negative and positive ends. Outliers were identified and removed to improve the model fit. However, this did not improve the overall fit considerably. Item 6 (family relationship) and item 7 (staff relationship) displayed lowest range and spread at logit 0. Item 13 (make choice) and item 12 (live with others) displayed the largest range. However, both of them exhibited differential item functioning: item 13 by age and item 12 by language group. As such, they were not considered further for health state classification. Item 4 (living situation) and item 8 (friendship) covered similar range, and neither exhibited differential item functioning. Conceptually, item 4 is better at measuring key characteristics of interpersonal environment and is therefore a strong candidate to use in the classification system.

#### Physical health

The Rasch analysis showed that item 1 (physical health) had better overall fit statistics compared to item 2 (energy). The person-item threshold distribution suggested that there are no item thresholds at the person locations at either negative or positive ends. Item 2 exhibited differential item functioning by gender and therefore was not considered further for the classification system. Item 1 was selected to represent the dimension.

#### Self-functioning

The response categories for two items were ordered on the logit scale. Item 11 (take care of self) was disordered. Rasch analysis of two items, item 9 (keep busy) and item 10 (do fun things), displayed good fit statistics. Item 9 covered a larger range than item 10; however, it exhibited differential item functioning by age. Therefore item 10 was chosen to represent the dimension.

### Final classification system

The final health state classification following the factor analysis and the Rasch models include the following five items: memory, mood, living situation, physical health and do fun things. Conceptually, the ‘memory’ item stands alone as a measure for cognitive decline. We felt that ‘mood’ was sufficient to represent a latent trait other than the three identified traits (physical, interpersonal environment and self-functioning). ‘Living situation’, ‘physical health’ and ‘do fun things’ were chosen to represent the three sub-scales of ‘interpersonal environment’, ‘physical’ and ‘self-functioning’. The items and response levels were developed into a dementia-specific health state classification system (and named AD-5D) and this is displayed in Table 5.

**Table 5:** Proposed health state classification system for the new preference-based instrument AD-5D

Dimension	Descriptions
Memory	You have **excellent** memory You have **good** memory You have **fair** memory You have **poor** memory
Mood	You have **excellent** mood You have **good** mood You have **fair** mood You have **poor** mood
Physical health	You have **excellent** physical health You have **good** physical health You have **fair** physical health You have **poor** physical health
Living situation	You have **excellent** living situation You have **good** living situation You have **fair** living situation You have **poor** living situation
Do fun things	You have **excellent** ability to do fun things You have **good** ability to do fun things You have **fair** ability to do fun things You have **poor** ability to do fun things

## Discussion

This is the first study undertaking a comprehensive dimensional and Rasch analysis of the QOL-AD to develop a dementia-specific health state classification system, the AD-5D. We performed exploratory and confirmatory factor analyses and Rasch analysis to investigate the latent factor structure and scaling properties of the QOL-AD and produce a classification system. From the factor analyses, we identified three multiple-item dimensions, named ‘interpersonal environment’, ‘physical’ and ‘self-functioning’ and two one-item dimensions (‘memory’ and ‘mood’). Through the iterative process of Rasch analysis, we found that the three dimensions of ‘interpersonal environment’, ‘physical’ and ‘self-functioning’ could be represented by three items (‘living situation’, ‘physical health’, and ‘do fun things’). With the inclusion of memory and mood, this results in a five-item health state classification system based on the QOL-AD instrument. These items cover the HRQL domains that are considered most relevant to people with dementia, including mood, global function and activities of daily living [16, 17]. The results of the Rasch analysis suggest that the QOL-AD has a level of validity for use in the assessment of HRQL in dementia, and therefore provides a strong base from which to generate a dementia-specific health state classification system. The results also support previous work assessing the psychometric acceptability of the QOL-AD for use in people with dementia and cognitive decline [25, 48].

This new health state classification system is the first step toward developing a preference-based instrument to measure HRQL in people with dementia from the QOL-AD. The next step is to undergo a valuation exercise to generate utility weights to produce a utility scale based on the preferences of the general population that can be used in the economic evaluations of healthcare interventions for people with dementia. This will be the first dementia-specific preference-based instrument based on the preferences of the Australian population, and the resulting utility scale will be used in the estimation of QALYs for dementia-specific interventions in a decision making process [34].

Our instrument is has some differences to other dementia-specific preference-based instruments, such as the DEMQOL-U [29] and the DQI [26], and further testing is required to understand the advantages and disadvantages of each. The DEMQOL-U does not include dimensions measuring ‘physical health’ and ‘skills in daily living’, and these may be considered relevant and important for people with dementia. The DEMQOL from which it is derived has not yet been widely used in clinical practice. The DQI is a 3-level 6-domain instrument that covers physical health, mood, memory, self-care, social functioning and orientation so there are some similarities in terms of item coverage, and further work should test the psychometric performance of both descriptive systems. Separately it will be important to test and compare the characteristics of the utility value sets which may differ due to the valuation methods used, and this may have implications for the QALY estimates derived from each instrument.

Following development of the utility scale for the AD-5D, there will also be the need to psychometrically test the values produced alongside those from the other dementia-specific measures and generic measures such as the EQ-5D. This could be done by assessing overlap in the constructs measured by the descriptive systems, and by determining the importance of the divergence in the constructs measured. This would enable us to understand the importance of dimensions that are not universal across all of the classification systems. If this analysis proves favourable, the AD-5D could be recommended for use in people with dementia, and has the potential to be widely used given that the QOL-AD is a popular instrument for use in people with dementia and cognitive decline [16].

In the economic evaluation of interventions and treatments for dementia the QALY, which focuses on HRQL, is the widely used metric. However, recent research has focused on the potential for using capabilities to measure the outcome of interventions, and this resulted in the development of the capability measure for older people (ICECAP-O) which measures capabilities such as attachment, security and control in older people [49, 50]. In measuring the outcomes of dementia interventions, the assessment of both HRQL and capabilities could be important, and therefore assessing the relationship between both types of measures could be informative, and result in a more holistic assessment of the impacts of dementia on the individual.

This study has limitations that should be considered. First, the data was collected using the QOL-AD nursing home version. While this version has been validated and widely used in trials and observational studies involving nursing home residents, there remain domain discrepancies between it and the (original) community-dwelling version. Within the scope of this study, we could not verify how well the new classification system represents HRQL dimensions in the QOL-AD community-dwelling version. Secondly, our sample was drawn from one single study in Australia, although participants were from 35 long-term care facilities. These participants may have certain characteristics and it would be useful to repeat the analysis on other samples. Thirdly, given that we used the self-report version, we do not know the extent to which the classification system is valid for the carer report version, and this is an area for further work. Fourthly, the results have not been validated on other samples as has been done in other health state classification system development work. Finally, the data did not contain information about the severity of dementia among participants. It was not possible to understand whether or not there were response differences by severity. We therefore do not know the extent to which the classification system is equally valid for different dementia spectrum, from mild to moderate and severe.

## Conclusion

This study proposes a new preference-based instrument (AD-5D) derived from the available health-related quality of life measure QOL-AD. The new classification system consists of five dimensions with four levels in each dimension. This is an important step in the future application of this commonly used HRQL measure for dementia in economic evaluations. Future valuation studies will enable this tool to be used to calculate QALYs in the economic evaluation of treatments and interventions for people diagnosed with dementia in any setting where the QOL-AD nursing home version has been used.

## Abbreviations

AQL-5D: Preference-based measure for Asthma Quality of Life Questionnaire
DEMQOL: Dementia specific health-related quality of life instrument
DEMQOL-U: Dementia specific health-related quality of life with preference-based measure
DQI: Preference-based instrument for dementia
DQOL: Dementia quality of life instrument
EORTC-8D: Cancer preference-based instrument
EQ-5D: EuroQol five dimensions questionnaire
HRQL: Health-related quality of life
ICECAP-O: Capability measure for older people
KHQ: King’s Health Questionnaire for urine incontinence
NICE: National Institute for Health and Care Excellence
PBAC: Pharmaceutical Benefits Advisory Committee
QALY: Quality Adjusted Life Year
QOL-AD: Quality of life for Alzheimer’s disease instrument
QOL-D: Quality of life questionnaire for dementia
